# Utilization, Delivery, and Outcomes of Dance/Movement Therapy for Pediatric Oncology Patients and their Caregivers: A Retrospective Chart Review

**DOI:** 10.3390/curroncol30070477

**Published:** 2023-07-06

**Authors:** Karolina Bryl, Suzi Tortora, Jennifer Whitley, Soo-Dam Kim, Nirupa J. Raghunathan, Jun J. Mao, Susan Chimonas

**Affiliations:** 1 Department of Medicine, Integrative Medicine Service, Memorial Sloan Kettering Cancer Center, New York, NY 10065, USA; brylk@mskcc.org (K.B.);; 2 MSK Kids, Memorial Sloan Kettering Cancer Center, New York, NY 10065, USA; 3 Department of General Internal Medicine, Memorial Sloan Kettering Cancer Center, New York, NY 10065, USA; 4 Department of Epidemiology and Biostatistics, Memorial Sloan Kettering Cancer Center, New York, NY 10065, USA

**Keywords:** dance/movement therapy, pediatric oncology, creative arts therapies, psychological distress, pain management

## Abstract

Children with cancer and their caregivers face physical and psychosocial challenges during and after treatment. Dance/movement therapy (DMT) has been used to improve well-being, promote healthy coping, and mitigate the impact of illness, but limited knowledge exists regarding DMT utilization, delivery, and outcomes in pediatric oncology. This retrospective study aimed to identify reasons for referral to DMT, DMT visit characteristics, key DMT techniques and processes, and clinician-reported outcomes. We examined the electronic medical records of 100 randomly selected pediatric patients (resulting in 1160 visits) who received DMT services between 2011 and 2021. Sociodemographic, clinical, and visit characteristics, referral reasons, and clinician-reported outcomes were reported as frequency and proportions. Qualitative thematic analysis was used to identify key DMT techniques and processes. Among 100 patients (63% female, aged 0–27 years), 77.9% were referred for psychological distress and 19.6% for pain. Two distinct DMT approaches were used during visits: a traditional DMT approach (77%) and a multisensory DMT approach (23%). The most common visit length was 15–25 min (41.6%), followed by sessions of 30–45 min (22.5%) and ≤10 min (18.1%). A total of 61.9% of DMT visits were inpatient and 38.1% outpatient. Of all visits, 8.8% were new and 91.2% were follow-ups. Caregivers were engaged in treatment in 43.7% of visits, and 5.5% of visits focused entirely on the work with the caregiver. DMT intervention focused on self-expression, emotional self-regulation, coping strategies, socialization, and caregiver–child interaction. Clinician-reported outcomes included enhanced coping with hospital experience (58%), improved pain management (27%), improved self-regulation (21%), and increased physical activation (13.2%). The results suggest DMT as a supportive intervention for psychological distress and pain management in pediatric oncology patients and provide insights into DMT practices and outcomes to guide intervention development and future research.

## 1. Introduction

Pediatric cancer, with an incidence of approximately 400,000 children and adolescents, is the leading cause of death in children worldwide [1,2]. In the United States, approximately 15,780 children (1 in 285) are diagnosed with cancer each year [3]. With pediatric oncology advances, survival rates for most childhood cancers have improved [4], but the psychosocial (e.g., anxiety, depression, difficulties in interpersonal relationships, non-compliance with treatment) and physical (e.g., fatigue, sleep disturbances, pain) burdens of cancer on patients remain high [5,6,7]. Furthermore, pediatric cancer also substantially affects the emotional and physical functioning of parents and caregivers [8,9,10,11,12]. Thus, standard supportive care plans should include interventions to manage cancer-related side effects and symptoms and to provide socio-emotional support [13].

Dance/movement therapy (DMT) is defined by the American Dance Therapy Association (ADTA) as “the psychotherapeutic use of movement to promote emotional, social, cognitive and physical integration of the individual” [14]. This therapeutic approach, grounded within a biopsychosocial framework, aims to support well-being and physical and social/emotional development, improve healthy coping, and decrease the impact of illness for children living with cancer and their caregivers. DMT therapists are master’s degree-level clinicians and licensed providers who utilize components of dance, improvised or structured movement, and creative and emotional expression, as well as other (psycho)therapeutic techniques (e.g., symbolism, metaphor), in a supportive therapeutic relationship within individual therapy sessions or group settings [14,15]. Movement and non-verbal behaviors are considered the primary mediums of assessment, interaction, and therapeutic interventions [16]. Several qualitative and theoretical contributions suggest that DMT can be implemented to support psychological adjustment [17,18], body image [19], and communication of difficult feelings and emotions [17,19]. DMT can also increase participation in therapeutic activities by reducing movement limitations in children and adolescents with cancer [19]. Results from two pilot studies suggest that DMT improves body image in adolescents with cancer [20] and quality of life for children receiving chemotherapy for brain tumors [21].

In medical settings, a distinct Multisensory Dance Movement Psychotherapy (MSDMT) approach is often incorporated to support the youngest patients during painful medical procedures [22,23,24]. This pain management approach to DMT is the application of pediatric medical dance/movement therapy with an added emphasis on the role of the body and multisensory experience to support physiologic and psychological coping, specifically related to medical illness. Within this approach, therapists provide children with a variety of activities that redirect focus away from pain and towards pleasurable sensory sensations [22,23]. These activities include the use of movement, music, touch, breath awareness, hypnosis, imagery, and meditation to augment pain control. One exploratory study examined pain control responses to MSDMT among pediatric neuroblastoma patients receiving an antibody therapy called 3F8 [25]. The study found that patients who were engaged, enthusiastic, had a capacity to develop coping skills, and were earlier in their treatment tended to have a positive pain control response to MSDMT. As such, MSDMT could be a noninvasive method that complements pharmacological and medical treatments.

Despite DMT’s long presence and great promise in medical settings, including oncology [23], little is known about DMT utilization, delivery, and outcomes I n pediatric oncology patients and their caregivers. Beyond two pilot studies [20,21] and several theoretical contributions [17,18,19,22,23,24,26,27], no data describe the therapeutic provisioning of DMT in this context. To address this critical knowledge gap, this study aimed to identify: (1) reasons for referral to DMT; (2) visit characteristics: DMT approach, session length, setting, visit type, and caregiver involvement; (3) key techniques and processes of DMT intervention; and (4) clinician-reported outcomes.

## 2. Materials and Methods

### 2.1. Study Setting

The Integrative Medicine (IM) Service at Memorial Sloan Kettering Cancer Center (MSK) has offered DMT since 2003, averaging 1000 inpatient and outpatient pediatric visits per year (approximately 42,120 visits since the program’s inception). Through dance and movement within a safe therapeutic environment DMT encourages patients to express their feelings and experiences, helps them to develop new coping and effective communication skills, supports child–caregiver relationships, and promotes body awareness, self-esteem, and socialization [22,23,24]. Dance/movement therapists also provide counseling, education (e.g., psychoeducation, education on services, development of coping strategies), and support to caregivers [23]. Within an inpatient setting, dance/movement therapists provide DMT individually to patients, together with caregivers, at the bedside, or in weekly group sessions in the pediatric recreation center. Within the outpatient setting, dance/movement therapists attend to patients and their caregivers while they are receiving or waiting for treatment. All IM services are offered free of charge.

### 2.2. Data Sources

Data were obtained from the electronic medical records (EMR) of pediatric in- and out-patients who received DMT services between January 2011 and December 2021. We employed a simple random sampling technique using a random number generator to randomly select 100 unique patients. Abstraction of 100 patient charts resulted in 1160 DMT visit notes, from which the following data were abstracted: sociodemographic characteristics (i.e., gender, date of birth, race, ethnicity), clinical variables (i.e., age at appointment, cancer type), referral reasons, DMT visit characteristics (i.e., intervention type, session length, setting, visit type), clinician-reported outcomes (as noted as clinical observations of change pre–post session in the visit notes), and key features and specific processes of DMT intervention. MSK’s institutional review board (IRB) approved the retrospective study protocol (IRB #17-481).

### 2.3. Data Analysis

To describe the sociodemographic and clinical patient characteristics, we calculated descriptive statistics, such as means and medians for continuous data and frequencies and percentages for categorical data. We used age-at-appointment categories for patients based on the Centers for Disease Control (CDC) Child Developmental Milestones [28]: “infants and toddlers” (0- to 2-year-olds), “preschoolers” (3- to 5-year-olds), “middle childhood” (6- to 11-year-olds), “adolescents” (12- to 17-year-olds), and “young adults” (18- to 39-year-olds). For race and ethnicity, we followed the Office of Management and Budget (OMB) standards [29]. All analyses were performed using SPSS version 29 [30].

To identify key features and specific processes of DMT intervention, we analyzed DMT visit notes using an inductive approach to thematic analysis [31,32]. To ensure inter-coder agreement, the first 10 visit notes were coded independently by 2 coders (KB and SDK), who then discussed and resolved discrepancies. This procedure was repeated 5 times, after which a formal codebook was developed to ensure the validity and consistency of the results. The remaining notes were coded by SDK, with the senior coder (KB) providing supervision for every 10 visit notes. After coding all 1160 visit notes, codes were grouped into themes, which were defined and reviewed with the study team. 

## 3. Results

### 3.1. Sociodemographic and Clinical Characteristics

As shown in Table 1, the mean age of patients in our sample was 8.24 ± 6.26 years, and most were female (63%). Among 100 patients, the majority were White (64%) and not Hispanic or Latino (83%). In terms of age, receipt of DMT services was almost equally distributed among preschoolers, middle childhood, and adolescent groups (26.1% vs. 27.4% vs. 22.3%, respectively), with fewer DMT visits delivered to infants and toddlers (16.5%) and young adults (7.7%). The most common pediatric cancer types were neuroblastomas (45%), followed by sarcomas (16%), leukemias (13%), and lymphomas (11%). Patients with blood and immune disorders received the most DMT visits (37 per patient, on average), followed by those with brain tumors, neuroblastomas, lymphomas, and leukemias (15, 12, 12, and 10 visits per patient, on average, respectively).

### 3.2. Reasons for Referral to DMT

Table 2 shows the most common referral reasons by visit type (new vs. follow-up) and setting (in- vs. outpatient). Psychological distress was the most common referral reason overall (*n* = 904, 77.9%) and across visit types (new visit: *n* = 74, 72.5%; follow-up: *n* = 830, 78.4%) and settings (inpatient: *n* = 670, 93.3%; outpatient: *n* = 234, 52.9%). Pain was the second most common referral reason overall (*n* = 227, 19.6%) and across visit types (new visit: *n* = 21, 20.6%; follow-up: *n* = 206, 19.5%) and settings (inpatient: *n* = 25, 3.5%; outpatient: *n* = 202, 45.7%). Other reasons included psychological and/or developmental support (*n* = 8, 0.7%), Enhanced Recovery After Surgery (ERAS) (*n* = 7, 0.6%), end-of-life care (*n* = 4, 0.3%), and fatigue (*n* = 2, 0.2%). No referral reason was specified in eight cases (0.7%).

### 3.3. Visit Characteristics: DMT Approach, Session Length, Setting, Visit Type, and Caregiver Involvement

Visit characteristics are presented in Table 3. Of 1160 visits, 102 (8.8%) were new visits, and 1058 (91.2%) were follow-ups. The most common session length was 15–25 min (*n* = 483, 41.6%), followed by 30–45 min (*n* = 261, 22.5%), and ≤10 min (*n* = 210, 18.1%). DMT was provided 718 times (61.9%) in the inpatient setting and 442 times (38.1%) in the outpatient setting. Caregivers were present 507 (43.7%) times during visits, and 64 (5.5%) sessions focused on caregivers (e.g., education, support). Traditional DMT (*n* = 893, 77%) was offered almost four times as often as MSDMT (*n* = 267, 23%).

### 3.4. Key Techniques and Specific Processes of DMT Intervention

Qualitative analysis, focusing on key techniques and processes of DMT, elicited four main themes. These themes are discussed below.

Theme 1. Self-expression and meaning-making. Dance/movement therapists create a safe therapeutic environment and encourage children to express themselves primarily through natural movement, employing techniques such as metaphorical representation, symbolism, or play. To support a sense of agency, therapists follow the child’s lead and tailor session activities (e.g., physical role-play, physical imagine-play, free dance, and choreographed dance) to their individual needs. Dance and movement offer a creative outlet for emotional release, and physicality provides a sense of control.

Theme 2. Emotional self-regulation, feeling identification, processing, and validation. Dance/movement therapists use techniques such as: (1) mirroring (e.g., embodiment or reflection) of the child’s physical expression or non-verbal communications; (2) attunement and rhythmic synchronizing to the child’s verbal and non-verbal physical and emotional state; and (3) other kinesthetic–sensory techniques (e.g., touch, sound) to assist children who under- or over-regulate to identify, physically express, process, and validate suppressed or difficult feelings.

Theme 3. Embodied coping strategies. Dance/movement therapists focus on embodied activities that increase children’s body awareness and help them recognize, understand, and respond to physical signs of distress. These activities include: (1) grounding techniques to slow down stress responses and emotional or physiological dysregulation; (2) anchoring to bring the patient’s attention to the present moment or shift sensations from anxious to calm (e.g., dancing to a favorite song); (3) auditory cues (e.g., entrainment) to redirect energy and attention toward positive, calm, and self-empowered emotional states; and (4) anxiety reducing activities (e.g., breathing exercises, embodied meditation, guided imagery) tailored to developmental and cultural preferences.

Theme 4. Socialization and caregiver-child interaction. Therapists teach caregivers how to read and respond to non-verbal (e.g., muscular tension, facial expression) and verbal cues (e.g., gurgling, babbling, crying) through movement (e.g., rocking) and use of props (e.g., toys, shakers) to support bonding and dyadic regulation with the youngest patients. With older patients, therapists engage caregivers in movement-based games, creative dance, expressive movement, or role-playing to help build responsive caregiver–child interactions and improve communication.

### 3.5. Clinician-Reported Outcomes

Clinician-reported pediatric outcomes are presented in Table 4. These included outcomes related to the treatment of psychological distress, such as enhanced coping with the hospital experience (*n* = 663, 58%) and improved self-regulation (*n* = 241, 21%), as well as improved pain management (*n* = 311, 27%) and increased physical activization (*n* = 151, 13.2%). Caregiver outcomes included decreased burden (*n* = 183, 16%) and enhanced parent–child relationship (*n* = 10, 0.9%). 

## 4. Discussion

In this retrospective chart review, we used quantitative and qualitative methods to analyze 1160 pediatric DMT treatments across 100 randomly selected patients. To the authors’ knowledge, this is the largest pediatric DMT study reported to date. We found that DMT can be successfully used across pediatric age groups, cancer types, and treatment settings to help treat psychological distress and improve pain management.

In our study, the most common reason for referral to DMT services was psychological distress, including anxiety, stress, and depression. This finding highlights the psychological and psychosocial effects of cancer diagnosis, hospitalization, and treatment on children [33]. These symptoms can significantly impact the quality of life, psychosocial development, symptom management, and treatment compliance [34], ultimately leading to lasting negative effects on patients’ physical and psychological health [35,36].

To target psychological distress, DMT therapists use dance and movement to help children express thoughts, emotions, and body sensations that are often difficult for them to verbalize. Movement used in sessions encompasses body postures, gestures, breathing exercises, natural and spontaneous movement, improvised dance, and various movement and dance sequences [37]. Movement also stimulates the imagination, enabling the creation and living of new experiences, promoting self- and body awareness, and enhancing self-efficacy. This is particularly important for cancer patients, as increased self-efficacy is linked to decreased psychological symptoms and increased self-care behaviors [38]. Furthermore, therapists use dance and movement to support the development of emotional self-regulation, which enables patients to recognize, name, and express a broad range of emotions and experiences [22,23,24]. As a result, patients can improve their psychological outcomes, such as anxiety, depression, or stress. Among older patients, this increased self-awareness can also lead to changes in habitual response patterns and a better understanding of their impact on themselves and their relationships with others [23]. Moreover, movement and dance in DMT promote physical activity and vitalization and therefore target anhedonia, apathy, and underactivity, which are common symptoms in children living with cancer.

In our study, we also found that DMT services were requested for pain almost as often as for psychological distress among pediatric outpatients, indicating that these patients often experience pain not only as a result of their illness but also due to diagnostic and therapeutic procedures often performed in outpatient settings [39,40]. Anxiety and depression are also significant factors contributing to ongoing pain in patients after cancer treatment [41]. Pain experienced by children with cancer can vary in type and severity [42,43], but is understood to be both a sensory and emotional experience [41]. In addition, unmanaged pain during cancer treatment can cause more psychological distress and post-traumatic stress for patients and their families [43]. 

DMT, and MSDMT specifically, can provide a non-invasive and complementary pain management treatment for pain and physical discomfort in pediatric patients [24]. These therapies teach children embodied coping strategies, such as relaxation, redirection from the experience of pain, and self-regulation, through dance and movement. Techniques used in these therapies also include breathing, working with muscular tension, and attunement to somato-sensorial sensations. Furthermore, we also found that the MSDMT approach was offered almost 25% of the time and resulted in improved pain management among 98% of outpatients, suggesting that, for younger children, more sophisticated therapeutic approaches may be needed to help with pain coping. MSDMT may be particularly beneficial for children who might lack the comprehension and ability to effectively respond to verbal interventions when experiencing pain. This therapeutic approach recognizes that children, irrespective of their age, inherently absorb information through multiple senses. Moreover, this sensitivity to multisensory input becomes more pronounced during challenging medical situations. During visits, activities are administered by layering specific sensory experiences through playful engagements, which, at first, distract the patient, then ultimately support them to reach a meditative state when in heightened arousal or perceiving pain [23,24]. Within MSDMT, therapists help the youngest patients achieve a self-regulatory state by attuning to the child’s multisensory input and co-regulating their reactions to the painful experience. This is achieved through a variety of activities that are conducted by gradually incorporating various sensory experiences into playful interactions. Initially, these experiences serve as distractions for the patient, but eventually they help the patient attain a state of meditation when they are experiencing heightened arousal or perceiving pain [24]. As such, DMT results in enhanced coping with the hospital experience, improved pain management, and improved self-regulation. 

Caregivers also play a crucial role in children’s pain experiences and are often included in pain treatments [44]. Studies have shown that there is a connection between how caregivers respond to their child’s pain and the severity of pain, functional disability, and other somatic complaints that the child experiences [45,46,47]. Depending on the child’s developmental stage, parents may also be essential to the treatment process, as their support is necessary for children to improve their adaptive pain management skills. Without their targeted support, it can be more difficult for children and adolescents to make progress in this area. DMT not only facilitates socialization but also caregiver–child interaction through creative dance-play with their children during treatment, procedure, or hospital stay. Notably, in our study, caregivers were present and engaged in almost 44% of DMT visits. In addition, 5.5% of visits focused solely on caregiver support, resulting in decreased caregiver burden and enhanced parent–child relationship support. The inclusion of caregivers in therapeutic interventions should be strongly considered while developing treatment and research protocols.

We acknowledge several study limitations. First, the retrospective nature of this study limits our ability to examine other factors that may be associated with utilization and delivery of DMT (e.g., patient/caregiver feedback, perception of treatment benefits, outcomes/symptomatic relief). Second, this study was conducted at a single academic cancer center; therefore, our sample may not be representative of other populations, so the generalizability of our findings may be limited. Third, in our study, patients were specifically referred to DMT services by their health care providers; therefore, clinician referral bias might confound our results. Fourth, we assessed outcomes as reported by the clinicians; therefore, it is possible that, while highly trained, the two DMT therapists in our study may have personal biases that influenced their treatment approaches and reported outcomes. Finally, our DMT program is supported by specific institutional support that may not be available in other settings, and therefore it may be difficult to implement in less supportive contexts. Despite these limitations, our study represents an important step towards understanding pediatric DMT utilization, delivery, and outcomes.

## 5. Conclusions

In this retrospective study of over 1000 treatments among 100 pediatric cancer patients, we found that DMT is commonly offered to patients who experience psychosocial and physical difficulties related to cancer treatment and hospitalization. We also discerned specific patterns of utilization (e.g., session length, average follow-up visits) and described key features and specific processes of DMT (e.g., how DMT is delivered and the ways dance and movement are used in a therapeutic context) and clinician-reported pediatric and caregiver outcomes (e.g., enhanced coping with hospital experience, improved pain management, decreased caregiver burden). Our results suggest that DMT can be successfully used across pediatric age groups, cancer types, and treatment settings to treat psychological distress and improve pain management. This knowledge is instrumental in intervention development and will help formulate hypotheses for future research aiming to enhance the effectiveness of DMT for children living with cancer.

## Figures and Tables

**Table 1:** Patients’ demographic and clinical characteristics.

Characteristics	Number of Patients/Inpatient	Number of Visits/Inpatient	Average Visit per Patient
**Total**	***n***	***n* (%)**	***n***
	100	1160	
**Gender**			
Female	63	685 (59.1)	-
Male	37	475 (40.9)	-
**Age at Appointment (years)**			
Mean (SD)	8.24 (6.26)	-	-
Median	7	-	-
**Age at Appointment categories (years)**			
Infants & Toddlers (birth–2 years)	25/15	191 (16.5)/141 (12.1)	9
Preschoolers (3–5 years)	26/14	303 (26.1)/124 (10.7)	12
Middle Childhood (6–11 children)	22/8	318 (27.4)/191 (16.5)	14
Adolescents (12–17 years)	18/11	259 (22.3)/163 (14)	12
Young adults (18–39 years)	9/6	89 (7.7)/71 (6.1)	10
**Race**			
White	64	656 (56.6)	-
Black	12	89 (7.7)	-
Asian	7	89 (7.7)	-
American Indian or Alaska Native	3	124 (10.7)	-
Other and Unknown	14	202 (17.4)	-
**Ethnicity**			
Not Hispanic or Latino	83	926 (79.8)	-
Hispanic or Latino	16	177 (15.3)	-
Unknown	1	57 (4.9)	-
**Cancer type**			
Adrenal tumors	2	8 (0.7)	4
Blood and Immune Disorders	5	129 (11.1)	37
Liver tumors	1	7 (0.6)	7
Neuroblastoma	45	534 (46)	12
Brain tumors	3	45 (3.9)	15
Leukemias	13	132 (11.4)	10
Lymphomas	11	159 (13.7)	12
Sarcomas	16	124 (10.7)	8
Sacrococcygeal Teratoma	1	2 (0.2)	2
Wilms’ tumor and other kidney tumors	3	20 (1.7)	7
**Treatment type**			
Chemotherapy	48	635 (54.7)	-
Immunotherapy	21	233 (20.1)	-
Surgery	16	63 (5.4)	-
Chemoimmunotherapy	9	136 (11.7)	-
Bone Marrow Transplant	6	93 (8.0)	-

**Table 2:** Referral reason.

Referral Reason	Total *n* (%)	New Visit *n* (%)	Follow-Up *n* (%)	Inpatient *n* (%)	Outpatient *n* (%)
Psychological Distress	904 (77.9)	74 (72.5)	830 (78.4)	670 (93.3)	234 (52.9)
Pain	227 (19.6)	21 (20.6)	206 (19.5)	25 (3.5)	202 (45.7)
Other ^1^	29 (2.5)	7 (6.9)	22 (2.1)	23 (3.1)	6 (1.4)

^1^ “Other” included: psychological and/or developmental support; Enhanced Recovery After Surgery; end-of-life care; fatigue; and not specified.

**Table 3:** Visit characteristics.

Visit Characteristics	Total *n* (%)	Inpatient *n* (%)	Outpatient *n* (%)
**DMT approach**			
DMT *	893 (77)	698 (60)	195 (17)
MSDMT **	267 (23)	20 (2)	247 (21)
**Session length**			
≤10 min	210 (18.1)	151 (13)	59 (5.1)
15–25 min	483 (41.6)	352 (30.3)	131 (11.3)
30–45 min	261 (22.5)	168 (14.5)	93 (8)
60 min	168 (14.5)	39 (3.4)	129 (11.1)
75 min	22 (1.9)	3 (0.3)	19 (1.6)
≥90 min	12 (1)	2 (0.1)	10 (0.9)
Unspecified	4 (.3)	3 (0.3)	1 (>0.1)
**Setting**			
Inpatient	718 (61.9)	-	-
Outpatient	442 (38.1)	-	-
**Visit type**			
New Visit	102 (8.8)	60 (5.2)	42 (3.6)
Follow-up	1058 (91.2)	658 (56.7)	400 (34.5)
**Caregiver involvement**			
Work with caregiver	64 (5.5)	44 (3.8)	20 (1.7)
Caregiver engaged in session	507 (43.7)	226 (19.5)	281 (24.2)

* DMT—Dance/Movement Therapy. ** MSDMT—Multisensory Dance/Movement Psychotherapy.

**Table 4:** Clinician-reported outcomes.

Outcomes	N * (%)
**Pediatric**	
**Psychological Distress**	
Enhanced coping with hospital experience	663 (58)
Increased social interaction	372 (32.5)
Reduced anxiety/stress/fear	169 (14.7)
Decreased levels of depression	117 (10.2)
Comfort/End of life care	5 (0.4)
Improved self-regulation	241 (21)
Feeling calm and relaxed	199 (17.4)
Improved self-regulation skills	42 (3.7)
**Pain**	
Improved pain management **	311 (27)
**Other**	
Increased physical activization	151 (13.2)
Active physical engagement	78 (6.8)
Supported developmental milestones (developmental and sensory stimulation)	73 (6.4)
**Caregiver**	
Decreased caregiver burden	183 (16)
Caregiver education	73 (6.4)
Psychological needs and disease-related issues assessment	39 (3.4)
Caregiver counseling (e.g., coping with emotional distress, parental adjustment support)	71 (6.2)
Enhanced parent–child relationship	10 (0.9)

* N reflects the number of times the outcome was reported in visit notes. Note that for each visit note analyzed, multiple outcomes could be reported. Outcomes reported were not always in line with referral reasons; e.g., a patient could have been referred for pain, but during the visit the patient could also report feeling depressed, and the therapeutic activities would be focused on alleviating feelings of depression in addition to pain management. ** Improved pain management was reported in 98% of the MSDMT visits (263 of 267 total visits).

## Data Availability

Data sharing is not applicable to this article.
